# The Development and Validation of an Outdoor Free Play Scale for Preschool Children

**DOI:** 10.3390/ijerph20010350

**Published:** 2022-12-26

**Authors:** Sixian Li, Qianyi Jiang, Chenyu Deng

**Affiliations:** 1 School of Education, South China Normal University, Guangzhou 510631, China; 2 State Key Laboratory of Cognitive Neuroscience and Learning, Beijing Normal University, Beijing 100875, China

**Keywords:** outdoor free play, scale development, preschool children

## Abstract

Children’s outdoor free play, which is characterized by intensive physical engagement and diverse social interactions, plays a unique role in early childhood development and education. However, existing scales cannot comprehensively measure children’s performance in outdoor free play. The research purpose of this study was to develop and validate an Outdoor Free Play Scale for Children-Preschool Version (OFPS-P) with good reliability and validity, in order to provide a practical tool for teachers to understand the level of children’s outdoor free play. Based on the review of existing scales of children’s play and the uniqueness of children’s outdoor free play, we developed a scale with 12 items and validated the scale with two samples of preschool children with exploratory (n_sample1_ = 140) and confirmatory (n_sample2_ = 241) factor analyses. Four factors were identified in this scale: physical fitness, approaches to learning, social interaction, and imagination. The results indicated good reliability and validity of OFPS-P, which can be used to evaluate preschool children’s performance on outdoor free play and to support teachers’ effective support in outdoor play activities in kindergartens.

## 1. Introduction

Play is fundamental for children’s learning. Children fully develop their cognitive, social, physical, and motor abilities through experiences, inquiries, and social interactions in play. But current attention is usually paid to young children’s indoor free play, either in practice or in research. In fact, outdoor free play activities have distinct characteristics from indoor play, especially in the value of developing children’s physical fitness, social interaction, imagination, and approaches to learning [1,2,3,4,5].

Based on the unique value of children’s outdoor play activities on early childhood development, the statutory framework of 2021 UK Early Years Foundation Stage (EYFS) legislated that early education institutions in the UK must set up dedicated outdoor play areas for children aged 0–5, or plan and conduct outdoor activities every day, weather permitting [6]. Similarly, Head Starts in the United States, Canada, Scotland, and others, have also mentioned requirements for outdoor play in their statements [7,8]. Therefore, outdoor play has become a very important form of learning activity for young children in kindergartens.

### 1.1. Characteristics of Outdoor Free Play

According to the Affordance Theory, outdoor play has three fields of consideration: (1) the field of interactive limitation, which refers to the space’s physical features affecting interactions, (2) the field of promoted interaction, which recognizes how participants perceive and predict the common activity in the space, and (3) the field of free interaction, which reflects the participants’ agency and decision-making [9]. This model explicitly took children’s initiative as a part of the field of free action, also recognizing that this initiative is formulated and activated within a social and cultural context [9]. In China, under the influence of Confucian culture, teaching methods tended to be structured and teacher-centered. In recent years, influenced by the theory of children’s learning and development, the values of early childhood education have been changing. Free play that highlights children’s initiative and autonomy was included in the quality assessment system of Chinese kindergartens [10,11].

Outdoor play is beneficial to children’s overall learning and development. It provides children with a variety of outdoor experiences and opportunities for active learning. Children’s outdoor free play has unique characteristics. Firstly, different from static indoor play activities, children’s outdoor free play is dominated by dynamic activities, so children enjoy a high level of physical activity [12], so that they can exercise motor skills more effectively. Usually, kindergartens provide a variety of outdoor materials, supporting children’s hands-on abilities, such as rope binding, material fixing, etc., which further promotes the development of fine motor movements [13]. Because of the speed and strength associated with dynamic activities, children often face more challenges in outdoor free play [14] and need to assess risks to themselves and their partners in playful activities, to learn to manage risks, and to think about how to protect themselves when playing safely [15,16].

The second unique characteristic is related to the value of outdoor play for children’s approaches to learning. In particular, outdoor free play has the potential to enhance children’s activity organization and planning, problem-solving, concentration, and initiative. Generally speaking, there are fewer restrictions on young children’s outdoor free play. Children are more likely to arrange and choose their own play content as much as possible under safe circumstances in outdoor free play compared with in other play activities [17]. In this relatively permissive environment, young children’s play generally has richer content [1] and longer duration [18], and demands more on their ability to resist distractions [19]. Therefore it is a good opportunity for young children to develop persistence and perseverance, and control impulses [20,21].

Outdoor free play is also believed to be more conducive to promoting children’s social development. In this process, language communication, learning, imitation, and negotiation between children are particularly important [22].

Last but not least, outdoor free play offers children more opportunities to show their imagination. Compared with the relatively closed and restrained indoor space, the outdoor play space is more open, free, and diverse, so that children have the conditions to make “bolder” attempts. Their imagination and creativity are more likely to be stimulated accordingly [23]. Studies have shown that in outdoor play, children are more likely to show high levels of symbolic ability [24], and are also more likely to play with the contents detached from imitation and life experience, showing more uniqueness and originality [25].

The flexible outdoor learning environment provides teachers with the opportunity to observe children’s behavior, and children’s spontaneous play provides teachers with rich information about children’s individual interests and learning styles [26]. If teachers can understand the above-mentioned uniqueness of children’s outdoor free play, they can give targeted guidance in the activities, so that the unique value of outdoor free play can be brought into full play. However, the reality is that although teachers agree that kindergartens need to give children the opportunity to play outdoors freely, they seldom regard it as a meaningful educational link, or have no idea about how to play the role of teachers as a “scaffolder”. The author believes that developing a scale that systematically reflects the ability of children in outdoor free play will help to understand the quality of outdoor play activities and furthermore provide targeted guidance.

### 1.2. Age and Gender Characteristics of Children’s Outdoor Free Play Activities

Children’s outdoor free play significantly develops as children age. For example, in terms of physical fitness, young children’s overall gross motor movement [27] and fine motor movement [18] develop with age. In terms of challenges, as they grow in age, young children show more risk-taking play behaviors accompanied by distinct, easily recognizable body language and facial expressions [28]. In terms of social interactions, the frequency, diversity, and depth of children’s peer interaction increase as children age, same for the amount of children’s language expressions and positive feedback [29]. In terms of approaches to learning, in outdoor sand play, it is found that children’s independence, coordination, and cooperation improve with age [30]. Children’s imagination becomes more complex with age [31].

There are also studies revealing early gender differences in children’s outdoor free play. Boys are found to prefer and are frequently engaged in outdoor play, whereas girls prefer indoor sedentary activities better [32]. Although there were fewer reports of gender differences in children’s social interactions, approaches to learning, and imagination in outdoor play, gender differences between boys and girls in outdoor play were found to be less consistent. Barbu [33], using Parten’s description of social participation, reported that in outdoor associative play, girls participated more frequently than boys at the age of 3–4, whereas at the age of 4–5, boys participated more frequently than girls. At higher levels of cooperative play, girls participated more frequently than boys at age 4–5, but boys participated more frequently than girls at age 5–6. The reason behind may be that the chase and play fighting and the superhero play in outdoor play, which boys enjoy better, are helpful for boys’ social development [34].

The characteristics of age and gender development of children in outdoor free play activities provide a basis for the development of a scale for evaluating children’s outdoor free play. If the scale can sensitively reflect and express the specific performance of children of different ages and genders in various aspects of outdoor free play, a more developmentally appropriate outdoor free play setting can be prepared by teachers.

### 1.3. Analyses of Existing Relevant Children’s Play Scales

There have long been studies on the development of children’s play and different scales are available to understand children’s play behaviors. However, the existing scales of children’s play are mostly developed based on children’s indoor play activities. Based on a systematic literature review, the following five children’s play scales are found to tap some of the features of outdoor free play activities.

The Observational System for Recording Activity in Children-Preschool Version (OSRAC-P) [35] provides a relatively comprehensive understanding of children’s physical fitness in outdoor activities. It includes eight observational categories, among which are activity level, activity type, and activity location. The activity level classifies children’s physical activity level into stationary or motionless, stationary with limb or trunk movements, slow-easy movements, moderate movements, and fast movements. However, it does not investigate other characteristics of children’s outdoor free play, such as imagination and creativity, sociality and approaches to learning, so it is not specialized enough to be used for assessing the development of children’s outdoor free play.

Peer Play Scale [36] is focused on the complexity of children’s interactions in play and the organization and comprehensiveness of their play through real-time observation of young children’s play. It classifies children’s play into five levels, from non-interactive parallel play to complementary and reciprocal group play, in order to better identify and measure children’s social participation in play [36]. However, it ignores the characteristics of energy release, imagination, and creativity in outdoor play, and does not include items evaluating children’s approaches to learning in outdoor free play.

Revised Knox Preschool Play Scale (RKPPS) [37] is a classic comprehensive evaluation system of play. It includes four dimensions: Space Management, Material Management, Pretense-Symbolic, and Participation [37]. However, it is still relatively insensitive to the unique characteristics of children shown in outdoor play, so it cannot fully assess children’s outdoor free play.

The Symbolic Play Scale [38] sorts the potential sequence of the development of children’s symbolic play and classifies the development of children’s symbolic play into ten stages. However, it does not address the unique physical and challenge characteristics of outdoor play activities, nor does it assess the approaches to learning in children’s play.

The Play Checklist [39] is an observation tool that helps teachers formulate appropriate play goals. It includes items such as pretending with objects, role-playing, interactions, verbalizations about play scenarios, verbal communication during a play episode, persistence in play, entrance to a play group, conflict management, support of peers, etc. The sequence of the development of children’s play is presented under each item. Unfortunately, this is still not a scale developed specifically for the development level of children’s outdoor play activities, so it cannot fully reflect the basic characteristics of outdoor play, and neither can it investigate the developmental factors of certain abilities.

Based on the review of the above-mentioned classic play scales for young children, we realized that none of them can systematically capture the characteristics of children’s outdoor free play and its unique value to children’s development. We believed that developing a specialized scale for children’s outdoor free play development would fill this gap. Therefore, the objective of this study was to develop and validate an Outdoor Free Play Scale in Children-Preschool Version (OFPS-P), so as to provide a practical, easy-to-understand, and easy-to-operate tool for teachers to better understand the development of children’s outdoor play.

### 1.4. Outdoor Free Play in the Chinese Context

The word “play” in Chinese characters is composed of “游” and “戏”. “游” is a type of free and unrestrained physical activity, and “戏” mainly refers to playful activities. However, under the influence of Confucian culture, the play of ancient Chinese children tended to be “peaceful and harmonious”; children barely reached their limits in outdoor physical exercises and competitive games, therefore the intensity of physical exercises was reduced. Later, under the influence of the Imperial Examination System, children’s play become strongly learning-centered or focused on intelligence improvements, sometimes even showed signs of “not playing but only studying” [40]. For a long period of time, in modern Chinese kindergartens, play was only regarded as a break between teaching activities, or a type of teaching methods. Teachers only paid attention to educational play (have children play with the teacher), and children’s free play was not considered as part of the curriculum [41].

Ministry of Education of the People’s Republic of China [42] demanded kindergartens to ensure that children have two hours of outdoor activities every day, including one hour of outdoor sports. In order to protect children’s right to play and reduce teacher-guided educational play, administrative departments of education in Guangzhou and other Provinces required that children’s free play activities carried out in kindergartens should be at least 40 min or an hour [43,44,45].

More than half of the kindergartens in China carry out outdoor activities for more than two hours per day [46]. Unlike many forest kindergartens in Nordic countries, the outdoor environment of kindergartens in China is still changing from the “sports-centered playground” in the past to the “play-centered playground” now [47]. There were more artificial environments and fewer natural environments; few kindergartens had a forest or rich natural environment [48]. The outdoor playground mainly consisted of outdoor sports equipment, including facilities such as slides, jungle gyms, swings, dart boards, [49], as well as some sand pools, plants, ponds, building areas and other activity areas [46]. Outdoor activities mostly included free play and sports activities [11]. Sports activities are educational activities organized by the kindergarten for the purpose of strengthening children’s physique, promoting physical development and basic movement development, and improving children’s health. They commonly take the forms of morning exercises, physical education classes, sports days, school trips, etc. In China’s kindergarten evaluation and management system, structural teaching activities carried out outdoors, such as science-related activities and museum visits, were not considered as outdoor activities, but in the category of collective teaching activities; only children-initiated activities and play were seen as outdoor activities [10].

In Chinese kindergartens, indoor free play is mainly carried out in specialized areas. Through arranging certain materials such as equipment, toys, props, tools and operating materials, teachers support and guide children’s operation, communication, exploration and expression and therefore promote children’s independent learning and development. However, due to the stereotypical beliefs and the behavioral-orientated education in Chinese kindergartens, which focused on learning, teachers often offer specific learning tasks and materials during the process of play. In practice, the common way is to radiate or infiltrate the theme and objectives of collective teaching into the play environment and activities [50]; puzzle materials are also common, such as chess, mathematics learning materials, and jigsaw puzzles. There is a lack of play initiative and behaviors like “I can play as I want” [51]. Therefore, indoor free play is more inclined to learning instead of playing. As a result, kindergartens in China are actively exploring outdoor free play, hoping to give children more opportunities to play independently. Therefore, supporting children’s learning and development in outdoor free play has become one of the most concerned topics in the reform and development of kindergarten care and education in China.

Based on the review of the above-mentioned classic play scales for young children and the unique scenario in Chinese kindergartens for children’s outdoor free play, we believed that developing a specialized scale for children’s outdoor free play development would benefit teachers’ organization and scaffolding in children’s outdoor free play and fill the research gap of a lack of scales to systematically capture characteristics of children’s outdoor free play and its unique value to children’s development. Therefore, the objective of this study was to develop and validate an Outdoor Free Play Scale in the Children-Preschool Version (OFPS-P), so as to provide a practical, easy-to-understand, and easy-to-operate tool for teachers to better understand the development of children’s outdoor play.

## 2. Methods

Two rounds of classroom observations were conducted to collect data for scale validation in the summer and fall of 2020.

### 2.1. Participants

A total number of 381 children from four kindergartens in Guangzhou, Guangdong Province, China, were selected to participate in this study. During the first round (Summer 2020, T1), we had 140 participants (*M_age_* = 59.54, *SD* = 9.278; *n_boys_* = 47.1%; *n_3-4y_* = 40, *n_4-5y_* = 53, *n_5-6y_* = 47) from two public kindergartens, one of which was relatively small, providing kids with small-sized play equipment to have the outdoor play in the limited space in the community (*n* = 72).The other was bigger with spacious and specialized outdoor playground, offering abundant play materials and various equipment (*n* = 68). The classroom observation data generated from these participants were used for item analysis and exploratory factor analysis (EFA).

There were 241 participants (*M_age_* = 51.68, *SD* = 9.850; *n_boys_* = 49.4%; *n_3-4__y_* = 76, *n_4-5__y_* = 86, *n_5-6__y_* = 79) from two kindergartens during the second round (Fall 2020, T2). One of them was a forest kindergarten (*n* = 117) with a large and rustic outdoor playground, and a large variety of play equipment and materials, with a tradition of outdoor activities. The other kindergarten owned a moderate-sized outdoor playground and only a few items of large-sized play equipment. Nevertheless, the variety of play equipment and materials was abundant. The data collected in this round were used for confirmatory factor analysis (CFA).

### 2.2. The Development of OFPS-P

Our research team first searched existing play scales and looked for references that reflected the characteristics of children’s outdoor free play to construct the initial scale items. The research team performed pre-observations in the two kindergartens which participated in the first round and recorded the process of children’s outdoor free play, so as to improve the clarity of item descriptions, and to produce tentative scale items. The research team also consulted early childhood education professionals (including researchers, principals, and teachers) for advice on the coverage and appropriateness of the selected items.

We then randomly selected a class from each of the three grades (junior, middle, or senior) from four kindergartens and recorded a 6-min play video of each kid. Children’s behaviors in outdoor free play was evaluated based on the recorded information. The observer can easily review and discuss the behaviors to seek accurate coding with video-taped play episodes, which is not feasible for on-site observation. Among these play videos, we randomly chose 20 video clips (120 min in total) to train two assessors in using the scale for evaluation. They could pause the video after every minute and take 30 s to score. The total score of children’s behaviors for each minute will be calculated. In case of hesitant or inconsistent scoring during this process, the two assessors watched the video again together and discussed with each other to determine the final score. The inter-rater reliability of the two raters was ranged from 76.5% to 96.3%. The two assessors discussed the disagreed items until agreements among all items of the scale were reached.

The data collection of T1 and T2 followed a strict protocol, and observations were conducted with the same procedure in all four selected kindergartens. Before the observation, researchers explained the relevant information to teachers and parents, guaranteed to protect the privacy of participants and obtained informed consents. Each child’s outdoor free play process was filmed for 6 min after the warming-up stage (around 5 min), with a break interval of 1 min. Based on our observations, children were able to demonstrate a comprehensive variety of play behaviors for evaluation in 6 min as they had been warmed up.

In the process of filming, if children showed signs of withdrawal, fear, resistance, etc., researchers stopped recording immediately. Two research team members were responsible for data collection, with one being responsible for filming and the other for writing field observation scripts to ensure that the observation procedures were compliance with the agreement. After that, the research team members adjusted the description of the scale items according to the video clips and the written field observation notes.

Based on the initial scale, two assessors rated the scale in the video clips of children’s play collected in the first round (140 play episodes, 840 min in total), and modified item descriptions based on the play situation in the video and field observation. Later, the observation data were used in item analysis and EFA to construct a tentative scale. After that, the research team rated children’s play behaviors as shown in the video clips collected in the second round with the tentative scale (241 play episodes, 1446 min in total). The resulting data were used in CFA to finalize the scale construct. The specific contents of the OFPS-P can be found in Appendix A.

### 2.3. Item Selection and Scale Structure

During the formulation of the scale structure, we consulted several classic play scales of relevant content. Four basic and unique characteristics of outdoor play, i.e., physical fitness, approaches to learning, socialization, and imagination, were generalized based on this review of scales. They therefore served as the basic dimensions to assess children’s outdoor free play in the scale. We also selected items from classic play scales for reference.

Items reflecting participants’ approaches to learning were composed based on Preschool Learning Behaviors Scale (PLBS) [52] and Children’s Approach toward Learning rating scale [53]. According to children’s characteristics of outdoor free play, we selected primarilyfour items: Attention, Problem Solving, Organization, and initiative. To assess participants’ physical fitness, we modified the items of Activity Level, Gross Motor, Manipulation, Construction, and Interest, from OSRAC-P [35] and RKPPS [37]: Gross Motor, Manipulation, and Challenge. Scale items related to socialization, i.e., items related to Language Expression and Social Participation, were formed based on Language, Cooperation, Interaction, and Peer Support items from RKPPS [37] and Peer Play Scale [36]. In the Imagination factor, we specifically consulted the items of Imitation and Dramatization, as well as the Article Pretend factor in The Play Checklist [39] and the Symbolic Play Scale [38] to compose items of Symbolization and Creativity. Through the steps above, the initial scale with 12 items and 4 dimensions was formed.

## 3. Results

### 3.1. Descriptive Statistics

The descriptive statistics of children’s performance in different dimensions were reported in Table 1.

### 3.2. Item Analysis

Extreme value analysis and homogeneity test were conducted to examine the items of the scale. Results of the independent samples t-test showed that all items qualified psychometric standards (r = 0.424–0.631, *p* < 0.01), therefore a total of 12 items remained for exploratory factor analysis [54].

### 3.3. Exploratory Factor Analysis (EFA)

EFA was conducted to modify and simplify the scale structure. Results indicated that the scale was qualified for factor analysis (KMO = 0.685, sig. = 0.000) [54]. We conducted PCA and extracted four factors with eigenvalues > 1; the variance explained in total was 72.12%, as shown in Table 2. We named these four factors as “Physical Fitness”, “Approaches to Learning”, “Socialization”, and “Imagination”.

### 3.4. Confirmatory Factor Analysis

Data collected from the second sample were used for CFA. Results of the goodness-of-fit test indicated a good model fit, as shown in Table 3 [55,56,57]. Item reliability, composite reliability, and construct validity of the scale are shown in Table 4. The value of Cronbach *α* = 0.905 and CR value of all scale dimensions exceeded 0.6, indicating a good internal consistency of the scale [58,59]. The construct validity included convergence validity and differential validity. In this study, we used average variance extracted (*AVE*) as the index of the convergence validity of the scale. The construct validity of the scale was tested by comparing the square root of *AVE* and the Pearson correlation between dimensions. Test results showed that the *AVE* was larger than 0.5 for all dimensions, and the correlations between the dimensions were less than the square root of the corresponding *AVE*, indicating that the scale had ideal construct validity [59].

### 3.5. Gender and Age Characteristics of Children’s Outdoor Free Play Performance

As shown in Table 5, the results of independent sample t-test with gender as independent variable, overall and each dimension score as dependent variables, showed that children of different genders had significant differences in Physical Fitness (t = −2.394, *p* < 0.05) and Socialization (*t* = 2.325, *p* < 0.05). Specifically speaking, girls showed a lower level of physical fitness but a higher level of social skills compared with boys.

To further understand the sensitivity of the scale to the performance of children of different ages in outdoor free play activities, we conducted a one-way ANOVA with age as the independent variable, and the overall and each dimension score as the dependent variables. In Table 6, results indicated that children of different ages have significant differences in all dimensions: Overall (*F_(2, 238)_* = 96.512, *p* < 0.01), Physical Fitness (*F_(2, 238)_* = 967.484, *p* < 0.01), Approaches to Learning (*F_(2, 238)_* = 18.526, *p* < 0.01), Socialization (*F_(2, 238)_* = 25.987, *p* < 0.01), and Imagination (*F_(2, 238)_* = 11.928, *p* < 0.01). Follow-up post hoc comparisons revealed that no significant differences were found between the socialization of 4–5 year olds and 5–6 year olds (*p* > 0.05), neither in the imagination dimension between 3–4 year olds and 4–5 year olds (*p* > 0.05).

## 4. Discussion

### 4.1. The Uniqueness of OFPS-P

Four fundamental aspects were identified in OFPS-P to understand children’s outdoor free play, namely Physical Fitness, Approaches to Learning, Social Interaction, and Imagination. Physical Fitness highlights the most outstanding contribution of outdoor play to children, namely, movements [17], thus making up for the neglect of physical ability among previous play scales. The previous play scales did not highlight the aspect of Approaches to Learning in outdoor play, although the outdoor free play is an excellent venue for children to develop skills related to approaches to learning. The specification of the dimension of Approaches to Learning in OFPS-P reflected the expectation of children’s ability and approaches to learning in kindergarten [60,61].

Compared with educational play, free play cultivates children’s social communication ability in authentic situations [62]. The dimension of social interaction can well assist teachers in evaluating children’s language expression, social participation, and communication strategies. Compared with the indoor environment with organized layouts, the outdoor environment, which is less structural, is more conducive to children’s comprehensive learning, imagination, and creation. The dimension of Imagination clearly highlighted this feature [63].

### 4.2. Supporting Children’s Outdoor Free Play with OFPS-P

There are only 12 items in OFPS-P, which captures children’s specific behaviors in outdoor free play activities and is not affected by the types of outdoor play. Teachers need to make sure that children have been in the state of outdoor play (about 15 min after the start of play), then focus on each targeted child for about 6 min, and rating them individually before they achieve an overall picture of these targeted children’s play performance in outdoor free play in the aspects of Physical Fitness, Approaches to Learning, Social Interaction, and Imagination. In this study, the assessors of our research team evaluated each child’s performance based on 6 min of the child’s play, and the statistical results showed good reliability (*α* = 0.905), therefore 6 min should be a good amount of time when applying this scale. Children’s strengths and weaknesses in outdoor play can easily be detected, which is important for teachers to figure out the follow-up actions in supporting children’s development. To improve the accuracy of rating, we also encourage teachers to film children’s play and rate while watching the replay. Through the observation of one or several groups of children in the class, teachers can also reflect on their beliefs in outdoor free play, modify the outdoor play environment, and create opportunities for outdoor free play. Therefore, the scale is of great significance to improve the quality of outdoor free play in kindergartens.

### 4.3. Educational Suggestions on Children’s Performance in Outdoor Free Play

Based on the results regarding the age and gender-related differences in the performance of children’s outdoor free play, we found that there was an overall improvement in the development of children’s play behaviors as they grew up. However, in certain dimensions, the age differences were not significant. For example, we found that there were no significant differences in social interactions between K2 and K3 children’s social interactions in outdoor play. We believe that this result is closely related to the composition of the outdoor areas of kindergartens in China. The outdoor areas of Chinese kindergartens are mostly composed of areas for sports equipment, sand and water play, construction play, and gardening. These areas are generally less structured and lack teachers’ special guidance on peer interaction and communication strategies [64]. Therefore, K2 and K3 children tend to play independently after acquiring basic operating skills, and are rarely engaged in cooperative or group activities. We also found that there were no significant differences in Imagination between K1 and K2 children. This may be related to a lack of experience in playing outdoors and using outdoor facilities, and a higher level of restrictions on outdoor play for younger children from teachers, for safety considerations [65]. These limitations reduce the possibility for children to express their imagination freely in play. In fact, some studies have suggested that imposing too many restrictions on children’s outdoor play would hinder their development [66]. Therefore, this result offered constructive suggestions for teachers on providing sufficient opportunities for young children’s exploration and play in the outdoor free play session.

Consistent with the previous results on gender differences, we found that in outdoor free play, boys’ physical fitness was significantly better than girls’, whereas girls’ social development was significantly better than boys’. However, we found that both boys and girls performed well in outdoor play in Approaches to Learning and no significant gender differences were found. This is inconsistent with the existing studies on children’s play performance in classrooms [67]. We considered that the lack of gender differences might be due to the fact that both boys and girls can enjoy the opportunities of selecting the play contents and partners in the outdoor play so that both of them can learn to be persistent and solve problems appropriately. In terms of the ability of imagination in outdoor play, no significant gender differences were identified either. This may be related to the relatively open outdoor space, the relatively limited outdoor play time, and the less structured play facilities in outdoor play. Due to the lack of guidance and support from teachers, children in outdoor play mainly focus on energy release, and the materials and time that are important for children’s development of imagination are relatively limited.

Based on this analysis of children’s age and gender differences in outdoor free play, it is suggested that teachers should better examine the possibility of developing children’s physical fitness, social interaction, approaches to learning, and imagination in outdoor free play, and offer specialized guidance to the weak developmental stages.

### 4.4. Limitations and Suggestions for Future Studies

This study is unique in the development and validation of OFPS-P, which is valuable in understanding children’s play performance in outdoor free play and to support teachers’ facilitation of children’s learning in outdoor free play activities. There are also limitations in this study. First of all, the participating kindergartens are all from urban areas, which might not be representative to reflect an overall scenario of outdoor free play in the country. However, the kindergartens selected were of different levels of facilities and support for children’s outdoor play; therefore the results still have great implications. We suggest that the follow-up studies include kindergartens from a more diverse background, including rural areas. Second, it is particularly important to note that the scale developed in this study is only applicable to children’s outdoor free play, but not children’s sedentary or non-play behaviors. Therefore, it is suggested that future studies investigate the various types of activities in outdoor play besides, so that children’s outdoor experiences can be better understood. Thirdly, although OFPS-P showed good reliability and validity from the item analyses and factor analyses, we have not conducted test-retest validation and this will be the next step of further validation. As this is one of the first scales particularly developed to understand children’s outdoor free play behaviors, teachers are encouraged to use OFPS-P in their daily teaching and to enhance their role in the outdoor free play activities. Future studies can further explore the role of teachers in children’s outdoor free play with the help OFPS-P and the way to integrate OFPS-P in teachers’ professional development.

## 5. Conclusions

As an important component of kindergarten activities, outdoor free play plays a unique role in children’s development [68]. However, outdoor free play is usually considered as a “burn-out” session for children, and teachers generally do not consider it significant for children’s development, especially when more attention is paid to children’s academic learning [69]. As a result, there is a lack of resources to support teachers in providing help and support for children’s performance in outdoor free play.

This study is one of the first to develop and validate a tool for evaluating preschool children’s outdoor free play. Item analysis, EFA, and CFA showed that OFPS-P had good reliability and validity. The sensitivity to the performance of children across different genders and ages in outdoor free play evidenced the discriminant validity of the scale. Therefore, we considered OFPS-P as a scale with good psychometric characteristics and it can be used to evaluate children’s outdoor free play in practice.

## Figures and Tables

**Table 1:** Descriptive Statistics (*n* = 381).

Dimensions and Items		Age 3–4 (*n* = 116)		Age 4–5 (*n* = 139)		Age 5–6 (*n* = 126)	
		M	SD	M	SD	M	SD
F1: Physical Fitness	Q1 Gross Motor	1.99	0.50	2.92	0.66	3.47	0.88
	Q2 Manipulation	2.11	0.81	3.24	0.64	3.67	0.82
	Q3 Challenge	1.47	0.69	3.10	0.68	3.58	0.71
F2: Approaches to Learning	Q4 Attention	3.31	1.23	3.93	1.11	4.14	0.90
	Q7 Problem Solving	3.98	1.49	4.33	1.11	4.72	0.78
	Q8 Organization	3.16	0.82	3.38	0.89	3.62	0.66
	Q9 Motivation	3.90	0.74	4.12	0.66	4.29	0.52
F3: Socialization	Q10 Language Expression	2.84	1.03	3.32	0.78	3.53	0.75
	Q11 Social Participation	3.41	0.78	3.70	0.57	3.76	0.46
	Q12 Communication Strategies	2.99	1.12	3.60	0.76	3.67	0.70
F4: Imagination	Q5 Symbolization	1.91	0.94	1.90	0.98	2.34	1.17
	Q6 Creativity	1.92	0.94	1.96	1.05	2.52	1.33

**Table 2:** Factor Loading of the Four Factors Identified in EFA.

Dimensions and Items		F1	F2	F3	F4
F1: Physical Fitness	Q1 Gross Motor	0.774			
	Q2 Manipulation	0.791			
	Q3 Challenge	0.866			
F2: Approaches to Learning	Q4 Attention		0.619		
	Q7 Problem Solving		0.744		
	Q8 Organization		0.826		
	Q9 Motivation		0.718		
F3: Socialization	Q10 Language Expression			0.846	
	Q11 Social Participation			0.660	
	Q12 Communication Strategies			0.878	
F4: Imagination	Q5 Symbolization				0.937
	Q6 Creativity				0.951
Eigenvalues		1.499	3.784	1.962	1.409
% of the variance explained		17.074	19.541	18.980	16.527

**Table 3:** Model Fit Index of OFPS-P.

Model	χ2	df	χ^2^/df	RMSEA	CFI	TLI	SRMR
Four-factor model	90.246	48	1.880	0.060	0.979	0.971	0.035

**Table 4:** Model Fitness Index Shown in CFA.

Dim.	Factor Loading	Item Reliability	Composite Reliability	Convergence Validity	Discriminative Validity				**α**
		R-Square	CR	AVE	F1	F2	F3	F4	
F1 Physical Fitness	0.821–0.923	0.674–0.853	0.914	0.779	**0.883**				0.852
F2 Approaches to Learning	0.640–0.868	0.409–0.753	0.869	0.628	0.388	**0.792**			0.803
F3 Socialization	0.909–0.954	0.543–0.742	0.929	0.868	0.231	0.592	**0.932**		0.821
F4 Imagination	0.737–0.861	0.827–0.910	0.859	0.672	0.484	0.723	0.583	**0.820**	0.938

Note. Under Discriminative Validity, the values in bold on the diagonal represent the square root of AVE and values outside the diagonal represent the correlation between dimensions.

**Table 5:** Boys’ and Girls’ Performance in Outdoor Free Play.

Dimensions	Gender (M ± SD)		t	*p*
	Girl (*n* = 122)	Boy (*n* = 119)		
Physical Fitness	8.29 ± 2.956	9.17 ± 2.757	−2.394	0.017 *
Approaches to Learning	15.43 ± 3.421	15.67 ± 3.203	−0.557	0.578
Socialization	10.63 ± 2.141	9.99 ± 2.129	2.325	0.021 *
Imagination	4.57 ± 2.189	4.22 ± 2.192	1.276	0.203
Overall	38.93 ± 8.12	39.05 ± 7.964	−0.12	0.905

Note. * *p* < 0.05.

**Table 6:** Outdoor Free Play Performance of Children of Different Ages.

		3–4 Years	4–5 Years	5–6 Years	F	Post Hoc Test
Physical Fitness	M	4.93	9.44	11.58	967.487 **	K1 < K2 < K3
	SD	0.574	0.889	1.277		
Approaches to Learning	M	13.89	15.79	16.89	18.526 **	K1 < K2 < K3
	SD	3.769	3.276	1.955		
Socialization	M	9.01	10.66	11.19	25.987 **	K1 < K2, K1 < K3
	SD	2.646	1.569	1.520		
Imagination	M	3.88	4.00	5.33	11.928 **	K1 < K3, K2 < K3
	SD	1.953	1.928	2.319		
Overall	M	31.72	39.9	44.99	96.512 **	K1 < K2 < K3
	SD	7.411	5.634	4.697		

Note. ** *p* < 0.01.

## Data Availability

The data presented in this study are available on request from the corresponding author.

## Appendix A

**Table A1:** Items of Outdoor Free Play Scale in Children-Preschool Version (OFPS-P)

**Item**	**Definition**	**Level Description**	**Explanatory Note**
Gross Motor	The complexity of children’s use of large muscles	1: Play with limb or trunk involvement in a simple way without shifting location, such as knocking, swinging, and/or kicking a subject while remaining still. 2: Translocate at a slow and easy pace, such as crawling and/or wandering. 3: Translocate at a moderate pace with more coordinated body movements, such as smoother walking, hopping, steady climbing, and/or jogging. 4: Translocate at a faster pace, displaying strength in actions and an increased level of balance. Show complex actions, such as skipping, and/or jogging while operating an object. 5: Translocate at a very fast pace, show great control of muscles and balance. Perform complicated actions with great strength such as galloping, somersaulting, and/or speeding while operating an object.	The performance of children who meet level 1 is: standing in place, only moving hands or feet or trunk, such as sitting in place and tapping things with their hands; standing in place and swinging; stand still and kick toys around. It should be noted that children need to stay in place within 6 min of observation and meet the above criteria to be rated as Level 1.
Manipulation	The flexibility and coordination shown by children when using small muscles.	1: Use easy and repetitive hand movements, such as hitting, dumping, and squeezing. 2: Use simple hand gestures of matching and comparing objects without further actions. 3: Control hand to conduct basic stable operation of tool and material with coordination of eyes, such as classifying, cutting, inserting small objects. 4: Control hand steadily to operate tool and material, show, increased fine motor control, good coordination of eyes and hands, quick and easy operation of tools, such as pulling and/or yanking an object. 5: Control hand steadily to operate tool and material operation, fulfill complex tasks with coordinated eyes and hands, such as using tools to make things, tying ropes and operating materials, etc.	The performance of children who meet level 2 is: keep the gesture of comparing two play materials, but show no behavior such as classification or splicing that changes the materials. It should be noted that children need to maintain this kind of performance within 6 min of observation to be rated as Level 2.
Challenge	The level of bravery, self-control, and risk assessment by children, as well as their pursuit and experience of pleasure	1: Engage in relatively safe play of no difficulty, risk-taking or challenge. No joy of success after completing challenges. 2: Engage in play of danger, for instance, uncontrolled tumbling of the body which is perceivably very dangerous. 3: Engage in play fighting, perceivably relatively dangerous. 4: Engage in play of certain difficulty and danger, within an achievable range. With some signs of nervousness during the process, and expressions of pleasure after completion. 5: Engage in play of relatively high difficulty, with a certain level of danger but still within an achievable range. Definite signs of nervousness during the process, and a high level of excitement after completion.	In Level 1, the standard of “no difficulty, risk-taking or challenge” is that children are not interested in the play and show bored expressions. In Level 2, the standard of “perceptibly very dangerous” is that children are completely unaware of the existence of the danger, rampage and get injured. In Level 3, the standard of “perceptibly relatively dangerous” is that children can control their own behaviors but lose control occationally when playing.
Attention	The ability of children to suppress interfering factors	1: When visual or aural stimulations unrelated to play occur, immediately stop the play or perform unrelated behavior. Focus on unrelated stimulations for long periods of time without returning to the play or abandoning it altogether. 2: When visual or aural stimulations unrelated to play occur, stop the play or perform unrelated behavior for a moment, but is able to resume play soon later. 3: When visual or aural stimulations unrelated to play occur, continue the play while looking in the direction of the unrelated stimulants intermittently. 4: When visual or aural stimulations unrelated to play occur, continue the play and only look in the direction of the unrelated stimulants once or twice. 5: When visual or aural stimulants unrelated to play occur, continue the play without regarding the unrelated stimulants at all.	If children pay attention to stimulations unrelated to play within 6 min of observation, they will be rated as Level 1. If children show immediate attention to stimulations unrelated to play, but can return to their own play within the time range, they will be rated as Level 2.
Problem Solving	The children’s display of determination in attempting to solve problems, overcome obstacles, and variations in strategy.	1: When faced with difficulties, problems, or manageable risks, give up the play immediately. 2: When faced with difficulties, problems, or manageable risks, immediately request help from teachers or peers. 3: When faced with difficulties, problems, or manageable risks, make an attempt, but immediately request help from teachers or peers if the attempt fails. 4: When faced with difficulties, problems, or manageable risks, make multiple attempts before abandoning and then request help from teachers or peers. 5: When faced with difficulties, problems, or manageable risks, make multiple attempts with variations in strategy, and persevere without giving up.	Children rated as Level 1 may say thing such as “I’m not playing. Forget it”.
Organization	The children’s ability to arrange and organize personal or group play	1: Have no goal or plan for personal or group play, stopping play or switching play across area after a short period. 2: Have basic direction for personal or group play, but without a specific goal or plan, i.e., can focus on a certain play area, but without certainty in what to do, with frequent switching of play content within the area. 3: Have basic goals for personal or group play, i.e., can have basic levels of assertion in play content, although may be orderless in action, and may express play goals verbally. 4: Have clear goals and detailed plans for personal or group play, can firmly carry out plans step-by-step, be ordered in action, and can assist leaders in group play with initiative in organizing play. 5: Have new ideas in the process of implementing play plans, which are merged back into and enriching the original plan. Can assume the role of leaders in group play, coordinate different suggestions from within the play, and clarify the respective goals of peers in play.	In Level 1, the standard of “a short period” is 30 s or less. In level 2, the standard of “frequent switching of play content” is to change play content in the same area within 30 s or less. In Level 3, the standard of “orderless in action” is that children’s behaviors may be slow, hesitant and stagnant.
Motivation	The children’s willingness to participate in play.	1: Have certain interests and curiosities in the surrounding things and people, but without the manifestation of play. 2: Can keep participating in play, but with shifting interests. 3: Have the willingness to continue play, but without enriching of theme or content of play, and possibly without facial expressions of joy or focus. 4: Have the willingness to continue play, with considerable commitment, constant enrichment of theme or content of play, and possibly with facial expressions of joy or focus. 5: Have strong willingness to play with great commitment, can answer or raise questions related to play. With exciting facial expressions like excitement and positive behavior as laughter, or with positive verbal and body language expressing excitement.	In level 2, children can maintain the state of play, but the theme of play changes.
Language Expression	The complexity of language used by children in conversation with others.	1: Have no verbal expressions in play, or express with gestures, or mumbling. 2: Express with a single word or two-word phrases. 3: Express with three-or-four-word phrases or simple sentences, have a back-and-forth conversation with peers or teachers, and possibly raise “what” and “why” type questions and may switch topic in reaction to the needs of listeners. Have the willingness to converse and express with language. 4: Express with phrases or sentences of more than four words, use simple musical language, or two or more simple sentences in succession. Converse two rounds or above in a back-and-forth way with teachers or peers, possibly with multiple questions, playing word games, and express personal thoughts and feelings in front of others with language or gestures. Communicate with peers in organizing play, express roles with language, and perform verbal deduction. 5: Express diverse language with varying sentence structures and relational terms, expand on details of play or reiterate complete sequences of events (including cause, process, and results), or conduct creative narration about the theme of play. Express humor and amusement through language.	In Level 4, the standard of “use simple music language” is that children’s speech has the change of pitch and rhythm.
Social Participation	The degree of participation of children in group play.	1: Idle, watch, and/or daze, and observe incidental events of interest or certain group play of children, with occasional questions or suggestions raised, but without participation in play. 2: Play alone, with different play materials from the peers around, and without any communication with them, or participate in play with teachers only. 3: Participate in play similar to peers around, with similar play materials, eye contact with the peers, and possibly brief physical or verbal interactions, but without group play with the peers. 4: Participate in group play with other children, with communication about mutual play activity, but without division and cooperation or mutual goals. 5: Participate in group play with other children with clear division of labor and cooperation, all revolving around the same play goal.	
Communication Strategies	The ability of children to communicate and resolve conflicts in play using social skills and strategies.	1: Remain silent and self-focused in play, and have social behaviors of poor adaptability, such as sudden pushing over others, robbing toys from other children, damaging other children’s work, and signs of rejection and avoidance when approached. 2: Communicate in play with basic socially accepted behaviors, although largely passive, such as requesting from peers following guidance of teacher, responding to requests from peers, or one-way attention towards others. 3: Communicate in play well with socially accepted behaviors actively, such as asking for permission before taking others’ possession, or expressing oneself in front of others. 4: Communicate in play with social skills proficiently and have plenty of pro-social behaviors such as taking turns, sharing, and helping others. 5: Communicate in play proficiently with a multitude of communication strategies, effectively solving problems in communication, promoting the development of play, all with a sense of responsibility.	In Level 4, “communicate in play with social skills proficiently” refers to “having more prosocial behaviors”. The standard of “more” is that there are two or more prosocial behaviors in the 6-min observation.
Symbolization	Children’s symbolic ability in play.	1: Only exhibit simple mechanical behaviors without explicit situational content or any play pretend with objects or language. For instance, pushing toy cars beeping around on the floor. 2: Display symbolized behaviors with self or objects, supported by real-life objects or close likenesses thereof. For instance, pretending to eat with chopsticks, or letting dolls eat with chopsticks. 3: Display symbolization behaviors of replacing one object with another, engage in social role-play with a certain level of plots. For instance, playing the role of firefighters driving wooden planks (fire engines) to the forest to put out fires. 4: Display symbolization behaviors without the aid of objects, like pretending to drink without a glass. Engage in social role-play, with more than two or complementary roles, for instance play as the doctor with the doll as the patient. Have relative richness and coherency in storytelling. 5: Display symbolization behaviors with double or multiple social role-play characters, for instance, playing the role of the doctor as well as the father, with rich and coherent storytelling, creating imaginary scenarios through language.	In Level 3, the standard of “engage in social role-play with a certain level of plots” is that children will determine story stage and their performance according to their own roles. In Level 4, the standard of “relative richness and coherence in storytelling” is that two or more story stages have been generated, and the plots are logical.
Creativity	The degree of separation away from imitation and real-life experience, for the content and expression of the children’s play.	1: Repetitive and rigid play behavior. 2: Simple imitative behavior or straight copying frequent occurrences in life, like imitating others’ sound, action and facial expression. 3: Imitative, real-life based behavior with additional individual thoughts, for instance, imitating horse riding with broomsticks in addition to pretend sweeping, and vending cakes in a bakery. 4: Have individual ideas mainly with less imitation, for instance, building an alien spacecraft alone. 5: Have a relatively high amount of creative play content surpassing reality, or use tools and materials in a multitude of ways. For instance, after building “cakes” with mud and sand, inviting other children to “pay” to stomp the “cakes”.	In Level 3, the standard of “additional individual thoughts” refers to the new functions, backgrounds or meanings that children give to their play on the basis of imitating others or referring to life experiences. In Level 5, the standard of “a relatively high amount of creative play content surpassing reality” is that children use real world materials to create play content that does not conform to the general meaning of reality, which is innovative.
